# White civilians’ implicit danger evaluation of police officers underlies explicit perception of police

**DOI:** 10.1186/s41235-021-00343-9

**Published:** 2021-12-20

**Authors:** Vincenzo J. Olivett, David S. March

**Affiliations:** Florida State University, Tallahassee, USA

**Keywords:** Police, Civilian, Danger, Threat, Valence

## Abstract

The role of implicit processes during police-civilian encounters is well studied from the perspective of the police. Decades of research on the “shooter bias” suggests that implicit Black-danger associations potentiate the perception of threat of Black individuals, leading to a racial bias in the decision to use lethal force. Left understudied are civilians’ possible associations of police with danger and how such associations pervade behavior and explicit views of the police. The current work begins to address this gap. In two within-subjects studies, we separately assess police-threat (i.e., safety/danger) and police-valence (i.e., good/bad) associations as well as their relative influences on explicit perceptions of police. Study 1 revealed that implicit threat evaluations (police-danger associations) more strongly predicted negative explicit views of the police compared to implicit valence evaluations (police-negative associations). Study 2 replicated these findings and suggests that individuals evaluate the police as more dangerous versus negative when each response is pitted against each other within single misattribution procedure trials. The possible implications for explicit attitudes toward police reform and behavior during police-civilian encounters are discussed.

**Supplementary Information:**

The online version contains supplementary material available at 10.1186/s41235-021-00343-9.

## Significance statement

Much research has focused on processes underlying racial bias in police officers’ decisions to use lethal force (e.g., research on shooter bias). Such work emphasizes Black-danger stereotypes and their role in potentiating a self-protective survival response (e.g., the shoot behavior). However, no work has considered how threat-related processes might manifest among civilians during encounters with police and how a danger association may pervade explicit attitudes and behaviors. That is to say, whereas the cognitive roots of police violence are relatively well understood, the cognitive fallout of systemic police violence is not. The current work begins to shed light on this issue by exploring the role of civilians’ implicit danger and valence evaluations in underlying explicit perceptions of the police. The unique contributions of police-danger versus -valence associations in underlying explicit views of the police are examined, and potential implications for behavior are discussed.

## Introduction

A large body of work exploring mechanisms underlying police attitudes and behavior during police-civilian interactions typically focus on anti-Black bias as a source of violent outcomes (e.g., Correll et al., 2007; Payne & Correll, 2020; Plant & Peruche, 2005). This work generally posits that stereotypes linking Black with danger underlie the “shooter bias” (a propensity in laboratory studies for participants [often including Police] to more frequently shoot unarmed Black compared to White men; Correll et al., 2002, 2007). It has been suggested that Black-danger and Black-weapon stereotypes drive the misperception of unarmed Black individuals as threats, resulting in survival-motivated responses (e.g., shoot). Given the possibility of deadly repercussions from negative police-civilian interactions, the police-civilian dynamic is often studied from the perspective of the “police.” Yet essentially no research has explored this dynamic—and the possible role of danger associations—from the perspective of the “civilian”. In the current work, we propose that just as police may (mis)perceive civilians as a survival threat due to preexisting danger associations, civilians may analogously perceive police as a survival threat due to established danger associations. From this perspective, whereas the cognitive roots of systemic police violence are relatively more understood, the cognitive fallout is not. Exploring how the processes underlying police-civilian interactions from the civilian perspective are influenced by perceptions of police as a threat highlights another important path by which police violence perpetuates wider systemic societal injustice.

From the civilian perspective, encountering an officer involves interacting with an armed and potentially dangerous individual. Not only is there knowledge that the officer is generally armed, but exposure to examples of police violence may lead to a police-danger association absent any personal experience. The importance of danger as diagnostic to survival may result in a danger association becoming a central underlying component of the summary attitude toward police (Olsson & Phelps, 2007). The current work explores this idea by examining the relationship between implicit associations with police and explicit evaluation of the police. In doing so, we distinguish between threat (i.e., danger) and nonthreatening-negative (subsequently, “negative”) associations, as these are distinct components of an attitude with unique influences on explicit perceptions (March et al., 2018a, 2018b). We expect that explicit police evaluation is largely driven by a police-danger relative to police-negative association. In two within-subjects studies, we separately assess police-threat (i.e., safety/danger) and police-valence (i.e., good/bad) associations as well as their relative influences on explicit perceptions of police.

## Danger associations and the police-civilian interaction

### The police perspective

Research on police-civilian interactions typically centers on the role of racial bias in officers’ decisions to use lethal force. Despite making up only 13% of the United States population, Black individuals represent 26% of police shooting victims, implying a racial disparity in the use of force (Payne & Correll, 2020). This pattern is studied in the laboratory using “shooter” type behavioral tasks (e.g., Correll et al., 2002; Greenwald et al., 2003; Plant & Peruche, 2005). In laboratory shooter studies, participants respond to Black or White, armed, or unarmed targets (or some cases, weapons or tools superimposed on Black or White faces; Plant & Peruche, 2005) by “shooting” armed and “not shooting” unarmed targets. Results reliably show a racial bias reflecting the disparity in police shooting; People tend to more quickly shoot armed Black than White targets and more frequently shoot unarmed Black than White targets.

Anti-Black bias in shooter-type tasks is hypothesized to be driven by stereotypes linking Black Americans to danger and weapons (Correll et al., 2007, 2015; March et al., 2020, 2021). Black-threat stereotypes are pervasive in the USA, as evidenced by trait ratings linking Blacks to criminality, hostility, and violence (Devine & Elliot, 1995). These stereotypes have been shown to influence visual processing and judgment (e.g., Donders et al., 2008; Duncan, 1976; Eberhardt et al., 2004; Payne, 2001), which may contribute to laboratory-based shooter bias. Highlighting the role of the Black-danger stereotype in decisions to shoot, increasing the accessibility of Black-danger associations increases shooter bias (Correll et al., 2007). Although most shooter studies utilize college student or general public samples, shooter biases have also been found in police populations (e.g., Correll et al., 2007; Plant & Peruche, 2005), though with less consistent results than non-police samples (Johnson et al., 2018). In sum, this research implies a link between danger associations and decisions to shoot.

### The civilian perspective

Relative to the amount of research on police-civilian interactions from the “police” perspective, little work has addressed this dynamic from the civilian perspective. The current work stems from the idea that automatic police-danger evaluations may drive downstream attitudes and responses to the police, which may ultimately influence civilian behavior in a police encounter. Analogous to the experience of a police officer encountering a potentially armed person (i.e., a danger), a civilian is likewise encountering a police officer who is almost certainly armed and dangerous (at least in the United States)*.* Moreover, the increased prevalence of videos depicting police violence against civilians may lead to social fear conditioned danger evaluations of the police, even absent any personal negative experience (Olsson & Phelps, 2007). Meaning, to a civilian, not only does a police officer encounter stereotypically involve exposure to an armed individual, but exposure to news and social media examples of police inflicting harm on civilians likely affect civilians’ attitudes (Campbell & Valera, 2020).

Exposure to media portrayals of police violence has been linked to negative evaluations of the police (Graziano, 2019) and a self-reported emotional fear response (Campbell & Valera, 2020). Conceptually similar results were found in a study measuring implicit associations linking police versus civilians to safety (e.g., comfort, peace, protection) versus fear (e.g., panic, concern, scared) on a word fragment completion task (Sargent & Newman, 2019). To White participants, police versus civilians were associated more with both personal safety *and* personal fear. The nature of both the safety (e.g., comfort) and fear (e.g., panic) words corresponds to a sense of personal safety. These findings likely reflect an association between police as a source of safety and as associated with feelings that accompany experiencing fear. That is, police may serve as both a source of safety from harm and are associated with, but not necessarily the source of, the accompanying fear emotion. That is, unlike previous results (Campbell & Valera, 2020), police here are not the source of fear (i.e., one is not afraid of the police), but instead police are a source of safety in fear-relevant situations. Indeed, neither fear nor safety associations related to explicit perceptions of police, implying that neither safety nor the associated fear emotion is central to explicit perception of police. Missing is a measure of police as the source of fear—the degree to which the police, as an attitude object, are perceived as dangerous. It is the police-danger association that we suggest has a primary role in underlying explicit perceptions of police.

## The current work

The current work measured White civilians’ implicit threat and valence evaluations of the police as well as their explicit attitudes toward the police.^1^ Two studies examined the degree to which people associated police, civilians, and uniformed non-police with danger and valence, whether those associations differed between prime types, and the degree to which those associations predicted explicit perceptions of police. Study 1 employed two misattribution procedures (MPs)—one with a safe/dangerous response dichotomy (threat) and one with a good/bad response dichotomy (valence). Study 2 builds on the results of Study 1 by pitting threat and negative associations head-to-head within a single MP, and additionally took into consideration the influence of police officers’ weapons. Prime conditions on each MP included images of police, civilians, uniformed non-police (e.g., firefighters). Explicit attitudes of the police were gathered using the Perceptions of Police Scale (POPS; Nadal & Davidoff, 2015). Our central hypothesis was that danger relative to valence primarily underlies explicit perceptions of police. Study 1 explored this question by testing the relative associations between distinct police-danger and police-negative evaluations as separate and simultaneous predictors of POPS responses. Study 2 explored the same question by testing the direction of the police-dangerous versus police-negative association in predicting POPS responses.

### Study 1

Misattribution procedures (MPs) are implicit measures that capture the strength of automatically activated affective or semantic content elicited by categories of prime stimuli (Imhoff et al., 2011; Payne et al., 2005; Payne & Lundberg, 2014). An MP trial typically entails a rapid presentation of a prime image or word followed by an ambiguous target (e.g., ideograms, often Chinese symbols). Participants are asked to ignore the prime and rapidly judge the target using keys that correspond to a response dichotomy (e.g., good vs. bad). The idea behind MPs is that participants misattribute affect or semantic content elicited by the prime to the target. Relative increases in one response over another after distinct prime categories serves as an index of the strength of the association between each prime category and each target response option. For example, White participants evaluate targets as less pleasant when preceded by Black than White primes, implying a stronger White versus Black-pleasant (or stronger Black vs. White-unpleasant) association (Payne et al., 2005).

In Study 1, White participants completed two MPs that captured associations between different categories of primes (i.e., police, civilians, uniformed non-police) and (a) safe versus dangerous or (b) good versus bad. The distinction between valence (good/bad) and threat (safe/danger) is relevant to the current work because danger is always negative, but negative is not always dangerous (March et al., 2017). Responses to danger versus negativity are driven by functionally distinct processes (March et al., 2018a, 2018b), and operationalizing police-negative without separately operationalizing police-danger associations prevents empirically distinguishing their relative strengths and influences. Capturing separate police-safety/danger from a police-valence association is critical to account for their unique role in underlying explicit police perceptions (March et al., 2020).

The danger versus valence (negativity/positivity) distinction implies that people may automatically associate the police with danger, safety, negativity, positivity, or combinations of each. For example, negative police-associations—perhaps associating the police with racial bias—does not necessarily mean that one associates the police with danger. Based on previous research (Sargent & Newman, 2019), we expect that police are indeed associated with safety. To the extent that police are also associated with danger, evaluations of targets following police primes may reveal a mixed pattern of safety and danger responses. Police may likewise be associated with positivity as sources of safety. But to the extent that they are also associated with negativity, evaluations of targets following police primes may reveal a mixed pattern of good and bad responses. We are agnostic as to whether the association between police and safety versus danger or good versus bad will be primary. Our main focus is on exploring the relative influence of the distinct valence versus threat associations on predicting explicit perceptions of police. Given the centrality of danger in attitudes and responses, it is our expectation that when considered simultaneously, police-danger versus police-negative will predict explicit perceptions of police.

#### Methods

One-hundred and thirty-eight White (*M*_age_ = 19.28, *SD*_age_ = 1.15) American undergraduates (108 female) participated for credit in a psychology course. Seven participants were excluded from some analyses due to missing one of the MPs (*n* = 5) or the POPS (*n* = 2)*.* The study was administered online via Inquisit (Millisecond Software). Participants were told that the study was designed to measure how people make rapid judgments. They were instructed that two images would flash very quickly one after another, the first being a typical picture and the second being a “Chinese character.” Participants were instructed to ignore the first image because it was part of a different version of the study and on each trial quickly judge whether they thought the Chinese character meant something “Good” (or “Safe) or “Bad” (or “Dangerous”). They completed a practice block and subsequently completed the two counterbalanced MPs. After finishing both MPs, participants completed the POPS and reported demographic information.

##### Stimuli

Thirty images were sourced from the Internet (10 police, 10 civilian, 10 uniformed non-police [e.g., firemen, postal workers]; see Additional file 1 for all stimuli). Prime images were cropped to 500 × 500 pixels and faces were blurred so to minimize responses to idiosyncratic characteristics that may not be consistent across prime conditions (e.g., attractiveness). Chinese ideograms (500 × 500 pixel) were used as targets.

##### Misattribution procedures

**Fig. 1:**
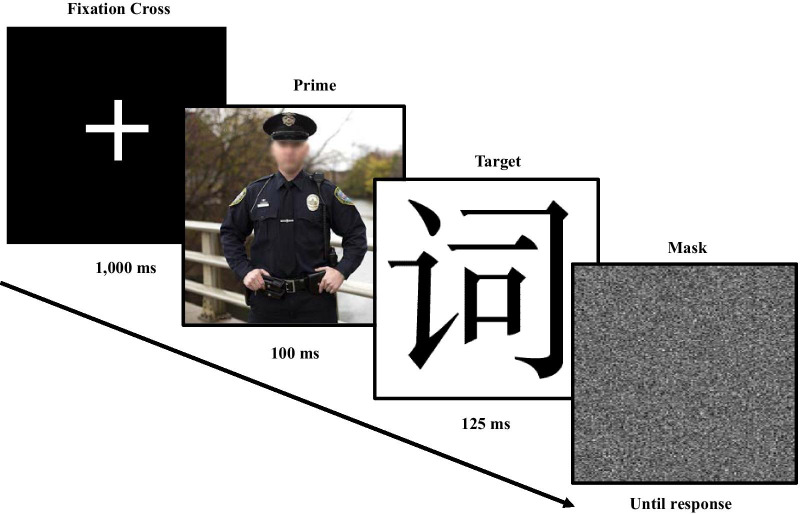
Depiction of a single misattribution procedure trial

Two MPs differentially measured implicit valence or threat evaluations. Each MP used the same prime stimuli and block/trial structure. During both MPs participants completed one block of 8 practice trials (consisting of neutral objects as primes) followed by a block of experimental trials.^2^ Each trial began with a 1,000 ms fixation cross, followed by a 100 ms prime image, a 125 ms blank screen, a 125 ms Chinese ideogram, and ended with a visual noise mask that lasted until participants entered a response (see Fig. 1).

Participants were told that they would first see an image followed by a Chinese character and that the character represented something “Good” or “Bad” for the valence MP or “Safe” or “Dangerous” for the threat MP. They were told to ignore the prime and to use the keyboard to rapidly indicate which response alternative the character represented. Ten images of each prime condition (10 police, 10 civilian, 10 uniformed non-police; 30 total critical trials) were randomized within the block. The choice of “good” versus “bad and “safe” versus “dangerous” within each MP was the dependent measure. Four-hundred and thirty-nine trials (2.67%) were removed from analyses due to response times (i.e., 3rd quantile; Tukey, 1977). Conclusions based on inferential tests and direction of effects are unchanged when excluded trials are retained.

##### Perceptions of police scale

The Perceptions of Police Scale (POPS; Nadal & Davidoff, 2015) served as a self-reported measurement of attitudes toward the police. The POPS consists of 12 statements about the police that capture the extent to which people see the police as trustworthy, reliable, unbiased, and responsible. For example, items include, “The police are good people,” “The police provide safety,” “Police officers treat all people fairly.” Participants respond to each item on a 5-point scale ranging from 1 = “strongly disagree” to 3 = “neither disagree nor agree” to 5 = “strongly agree”. See the Additional file 1 for the full scale.

##### Political orientation

Participants responded to the question, “What is your political orientation?” with answer choices “1 = extremely conservative, 2 = very conservative, 3 = conservative, 4 = neither conservative nor liberal, 5 = liberal, 6 = very liberal, 7 = extremely liberal” so that we could control for political orientation, which shapes civilians’ explicit evaluations of police (e.g., Brown, 2020).

#### Results

##### Good versus bad and safe versus dangerous comparisons within prime conditions

Responses were coded as 0 when a response of “good” of “safe” was entered, and 1 when a response of “bad” or “dangerous” was entered. Response values for each prime condition were averaged for each participant for both MPs, respectively. Values closer to 0.5 indicate evaluative ambiguity, values higher than 0.5 indicate a relatively more bad or dangerous association, and values below 0.5 indicate a relatively more good or safe association. Zero-order correlations among all variables are available in the Additional file 1.

**Fig. 2:**
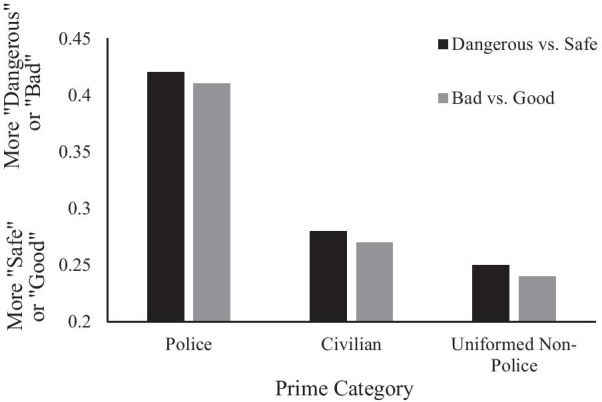
Mean dangerous versus safe and good versus bad evaluations by prime type

To examine whether police, civilian, or uniformed non-police were rated as more safe versus dangerous and more good versus bad, average responses within each prime condition were compared to 0.5 in a series of two-way paired-samples t-tests (see Fig. 2). For the safety versus danger block (*N* = 136), targets were evaluated as more safe versus dangerous following police (*M* = 0.42, *SD* = 0.34), *t*(135) = −2.61, *p* = 0.0100, civilian (*M* = 0.28, *SD* = 0.24), *t*(135) = −10.44, *p* < 0.0001, and uniformed non-police primes (*M* = 0.25, *SD* = 0.22), *t*(135) = −13.23, *p* < 0.0001. For the good versus bad block (*N* = 135), targets were evaluated as more good versus bad following police (*M* = 0.41, *SD* = 0.32), *t*(134) = −3.45, *p* = 0.0007, civilian (*M* = 0.27, *SD* = 0.22), *t*(134) = −11.67, *p* < 0.0001, and uniformed non-police primes (*M* = 0.24, *SD* = 0.23), *t*(134) = −13.34, *p* < 0.0001.

##### Valence and threat comparisons between prime condition within MP

To test whether White Americans differed in their associations between threat and valence by prime type, we entered (*N* = 133 participants who had both MPs) responses into a 2 (MP type: Valence, Threat) × 3 (Prime: police, civilian, uniformed non-police) multivariate repeated-measures ANOVA. A main effect of prime type, *F*(5,128) = 82.77, *p* < 0.0001, was not moderated by MP type (i.e., MP type x Prime), *F*(5,128) = 1.32, *p* = 0.26. Planned contrasts between prime types revealed that, within the threat MP, targets were evaluated as more dangerous following police than civilian, *F*(1,132) = 17.73 *p* < 0.0001, and uniformed non-police primes, *F*(1,132) = 29.31, *p* < 0.0001, and the latter two did not differ, *F*(1,132) = 2.43, *p* = 0.122. Within the valence MP, targets were evaluated as more bad following police than civilian, *F*(1,132) = 17.72, *p* < 0.0001, and uniformed non-police primes *F*(1,132) = 30.78, *p* < 0.0001, and the latter two did not differ, *F*(1,132) = 3.33, *p* < 0.07, (see Fig. 2).

##### Valence and threat as predictors of explicit attitudes

Our hypothesis relevant test is of the relative influence of valence and/or threat in underlying explicit perceptions of police. One-hundred and thirty-two participants had both MP and POPs data. To examine the relative influences of valence and threat evaluations of the police on explicit views of the police, in three separate models, we regressed POPS onto (1) valence evaluation, (2) threat evaluation, and (3) valence and threat evaluation, simultaneously, controlling for person-centered political orientation. Valence MP responses predicted POPS scores, *b* = −0.85 *t*(131)  = −.89 *p* < 0.0001, such that more bad implicit evaluations predict less favorable self-reported perceptions of police. Threat MP responses predicted POPS scores, *b* = −1.02, *t*(131) = −6.40, *p* < 0.0001, such that more dangerous implicit evaluations predict less favorable self-reported perceptions of police. However, when regressing POPS onto both threat and valence responses, simultaneously, whereas threat MP responses continued to predict less favorable POPS scores, *b* = −0.8447, *t*(131) = −3.98, *p* = 0.001 valence MP responses did not, *b* = −0.2695 *t*(131) = −1.22, *p* = 0.2235 (see Table 1). This implies that explicit POPS responses are underlied more by danger than negative associations.

**Table 1:** Results of regression analyses in Study 1 predicting POPS

Predictor	Model 1		Model 2		Model 3	
	*b*	*t, p*	*b*	*t, p*	*b*	*t, p*
Good/bad	− .85	− 4.89, < .001	–	–	− .27	− 1.22, .224
Safe/dangerous	–	–	− 1.02	− 6.40, < .0001	− .85	− 3.98, < .001

*b* represents unstandardized regression coefficients

#### Discussion

Police were evaluated as more safe than dangerous and more good than bad. Yet police were implicitly evaluated as more dangerous and more bad than civilians and other uniformed non-police. Together these results imply that although police are more associated with danger and negativity than civilians and uniformed non-police, the summary attitude of police reflects positivity versus negativity and safety versus danger. Study 1 supported our main hypothesis that danger over negative evaluations of the police more strongly predicted explicit views. That is, increased police-danger associations primarily underlied self-reported views of the police.

Two limitations arise in our assessments of threat and valence evaluations of the police. First, although participants completed two MPs that separately assessed threat and valence evaluations, these measures were highly correlated (*r* = 0.73, *p* < 0.0001). Danger evaluations in our study therefore may have captured some degree of negative associations, in line with our earlier supposition that danger is always negative. And given the non-significance of negativity in predicting POPS when considered simultaneously with danger, negative evaluations may have been driven by a danger association (March et al., 2018a, 2018b). Although assessing danger versus negativity separately allowed us to test their unique influence, Study 2 provides an alternative assessment of their relative influence by directly pitting danger and negativity head-to-head. Second, because police officers in the United States are nearly always armed, police in the prime images were equipped with visibly holstered guns. Weapons are a well-studied threat (Blanchette, 2006), and therefore officers’ weapons, not officers per se, may serve as a source of danger associations. Study 2 addressed this limitation by testing the negativity versus danger evaluations within trials containing both armed and unarmed police primes.

### Study 2

By assessing valence and danger in separate measures, Study 1 demonstrated that implicit danger evaluations are the primary predictor of explicit views. Left untested is the relative strength of danger versus negativity, that is, how individuals evaluate the police when negative and dangerous are pitted against each other in head-to-head trials, and the implications of these implicit associations for explicit perceptions. It is also possible that Study 1 results are driven by a weapons effect, that is, the presence of a weapon on the officer influenced implicit evaluations of the police. Study 2 addressed these issues by (1) changing the labels of the misattribution procedure to “Negative” versus “Dangerous,” and (2) including both armed and unarmed police officers. Procedures were identical to Study 1 with the exception that participants only completed one MP with a “Negative” versus “Dangerous” response dichotomy before filling out the POPS. Given the findings of Study 1, it is expected that (both armed and unarmed) police will be evaluated as more dangerous than negative, and that relatively more dangerous (vs. negative), rather than more negative (vs. dangerous), associations will predict POPS responses.

#### Methods

Eighty-one White (*M*_age_ = 19.55, *SD*_age_ = 1.71) American undergraduates (67 female) participated for credit in a psychology course. The procedure and instructions were identical to Study 1, except participants only completed a single MP in which they were instructed to judge whether characters meant something “Negative” or “Dangerous” prior to completing the POPS.

##### Stimuli

Three prime conditions—police officers, civilians, and uniformed officials—were used in Study 2. Primes included 30 unique images. There were 10 civilian and 10 uniformed non-police prime images. Also, of interest was whether dangerous or bad evaluations of the police were a consequence of the presence of guns. Therefore, within the set of 10 police primes, five images showed police with visible firearms, and five images showed police in the absence of firearms (see Additional file 1 for all stimuli). Images were sourced from the Internet, cropped to 500 × 500 pixels, and included blurred faces. Chinese ideograms served as targets.

##### Misattribution procedure

The MP was designed to test the threat versus negative association within a trial. Trial structure was identical to Study 1 (1,000 ms fixation cross, 100 ms prime image, 125 ms Chinese ideogram, visual noise mask until response), except that upon the presentations of the ideograms, participants were instructed to rapidly make key responses indicating whether the symbol represented something “Negative” or “Dangerous.” The prime conditions were police officers (both armed and unarmed), civilians, and uniformed non-police. Stimuli were presented in random order twice within a single 60 trial block. One-hundred trials (2% of total trials) were dropped from analyses due to response times (i.e., 3^rd^ quantile; Tukey, 1977). Conclusions based on inferential tests and direction of effects are unchanged when excluded trials are retained.

#### Results

##### Negative versus dangerous comparisons within prime condition

Responses were coded as 0 for “Negative” and 1 for “Dangerous.” Responses near 0.5 therefore indicate evaluative ambiguity, with values higher than 0.5 indicating relatively stronger danger than negative evaluation. Response values following each prime condition were averaged for each participant. As there was no difference in negative versus dangerous evaluations of armed (*M* = 0.56, *SD* = 0.26) and unarmed police (*M* = 0.54, *SD* = 0.24), *t*(80) = 0.84, *p* = 0.41, suggesting that the presence of firearms is not driving the police-threat association, we collapse police into a single prime condition in subsequent analyses. Zero-order correlations among all variables are available in the Additional file 1.

To examine whether police, civilian, or uniformed non-police were rated as more negative versus dangerous, average responses within each prime condition were compared to 0.5 using two-way paired-samples t-tests. Targets were evaluated as more dangerous than negative after police primes (*M* = 0.55, *SD* = 0.23) *t*(80) = 2.03, *p* = 0.0458, more negative than dangerous after civilian primes (*M* = 0.39, *SD* = 0.20) *t*(80) = −4.87, *p* < 0.0001, and equally negative versus dangerous after uniformed non-police primes (*M* = 0.46, *SD* = 0.18) *t*(80) = −1.83, *p* = 0.0714.

##### Valence and threat comparisons between prime conditions

**Fig. 3:**
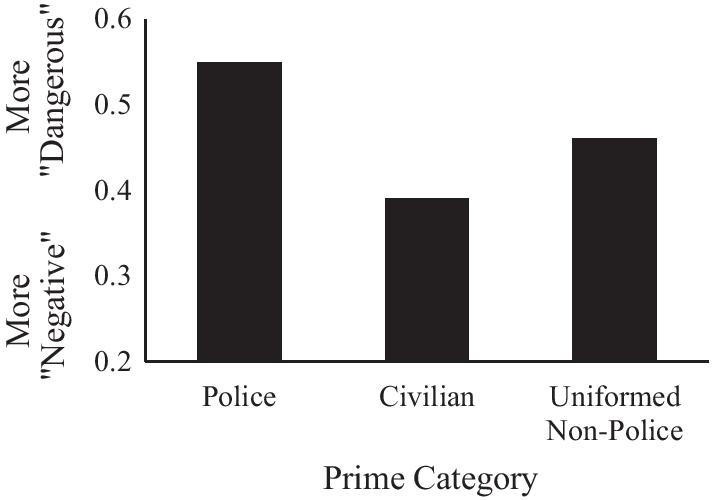
Mean danger versus negative evaluations by prime type

To test whether White Americans differed in their danger versus negativity associations as a function of prime type, we entered MP responses into a one-way repeated-measures ANOVA predicting the three-level prime type. A main effect of prime type, *F*(2,79) = 9.33, *p* = 0.0002, suggested varying danger relative to negative associations between prime types. A series of planned contrasts revealed that targets were evaluated as more dangerous following police than civilian, *F*(1,79) = 18.50, *p* < 0.0001, and uniformed non-police primes, *F*(1,79) = 8.45 *p* = 0.0047, and following uniformed non-police than civilian primes, *F*(1,79) = 9.42, *p* < 0.0029 (see Fig. 3).

##### Negative versus dangerous as predictors of explicit attitudes

Our hypothesis relevant test is of the relative influence of negativity versus danger in underlying explicit perceptions of police. To examine this, we regressed POPS onto MP response controlling for person-centered political orientation. Danger versus negative responses predicted POPS, *b* = −0.8565, *t*(80) = −2.87, *p* = 0.0053, such that more police-danger (vs. police-negative) responses underlie less favorable explicit views of the police.

#### Discussion

Study 2 revealed that participants implicitly evaluated the police as more dangerous compared to negative and as more dangerous than civilians and non-police uniformed professionals. Further, images of armed police officers were not evaluated as significantly more dangerous than images of unarmed police officers, suggesting that the weapons effect was not driving police-danger associations. Study 2 replicated results from Study 1 in supporting our main hypothesis: stronger police-danger versus negative association predicted more negative explicit views of the police. That is, the more police evoked danger relative to negativity, the more negative were self-reported views.

## General discussion

Decades of research on the “shooter bias” detail the role of implicit bias in driving police behavior during police-civilian encounters (Payne & Correll, 2020). Despite this, almost no research has examined similar processes from the perspective of civilians (cf. Sargent & Newman, 2019). The present work began to address this gap by assessing civilians’ implicit threat and valence evaluations of the police, as well how each process predicts explicit perceptions of the police. Study 1 demonstrated that implicit police-danger associations predict explicit views of the police over and above police-negative associations. This pattern was replicated and extended in Study 2 as increases in police-dangerous relative to negative associations predicted more negative explicit perceptions of the police. Together these findings imply that, at least among White Americans, direct or vicarious exposure to police violence (as possible sources of police-danger associations) may have unique implications for summary attitudes about the police. This is unsurprising given the concurrent rise in media coverage of police violence (Mehta, 2020) and calls for police reform (Robinson, 2020). Importantly, the observed relationship between implicit police-danger associations and less favorable explicit police evaluations does not imply that the former causes the latter. Instead, we suggest only that the automatic police threat versus negative association is a primary component of the explicit summary police evaluation.

### Behavioral implications from the civilian perspective

Imagine how you would react if you encountered a person with a gun running toward you. Imminent survival threats—such as armed individuals (or other predators)—activate the defensive survival circuit leading to rapid physical and psychological self-protective responses (LeDoux, 1996). Driven by an ingrained motive to survive, humans and non-human animals respond to threat by automatically recoiling (i.e., flight), engaging (i.e., fight), or pausing to hide or gather more information (i.e., freezing; Löw et al., 2015). As you may have imagined, confronting an armed individual may result in quick and reflexive physical protection movements (e.g., flinch), a desire to remove oneself from the presence of threat (e.g., run), or perhaps attempt to engage the threat in a self-protective fashion.

Consider these behaviors in the context of a police-civilian encounter. One way to understand the “shoot” behavior in biased shooter task performance is as an instance of automatic defensive behavior. By this view, Black-threat stereotypes potentiate (mis)perceptions of threat, which give rise to defensive behaviors (i.e., “shooting”). Indeed, officers often describe shooting as a “split-second” decision driven by a survival instinct. However, given that our data imply a primary role of danger evaluations of police among civilians, it is reasonable to suspect that civilians may evince analogous defensive behaviors during police-civilian encounters. After all, from the civilian perspective, these encounters involve a person approaching who is also holding a gun. That is to say, avoidant or non-compliant behaviors during arrest are perhaps related to automatic threat evaluations driven by police-danger associations. Though plausible, this claim lies outside the explanatory scope of present work, which was confined to demonstrating a unique relationship between police-danger associations and summary explicit summary attitudes. As we speak to below, examining the potential link between these associations and defensive behaviors and physiological responses is a key focus for future research.

### Limitations

Although we focused on police-danger versus negativity associations in White participants as a conservative test of our idea, this lack of diversity is a limitation. Notably, Black (and other, e.g., Hispanic) individuals are the disproportionate victims of police violence (DeGue et al., 2016; Edwards et al., 2019; Schimmack & Carlsson, 2020), and therefore may experience greater direct and vicarious fear conditioned police-danger associations (Olsson & Phelps, 2007). Given this, we suspect our results would extend also to other races and ethnicities of American individuals; indeed, the implicit police-threat evaluation and their ostensible physiological and behavioral implications may be stronger among populations more directly impacted by police violence.

Supporting this is research on mental representations of police faces (derived from reverse-correlated facial composites), wherein facial composites depicting Black versus White participants’ mental representations of police officers were rated as less good, more bad, and more dominating (i.e., potentially threatening) by both predominantly White and racially heterogenous samples (Lloyd et al., 2020). Moreover, when these composites were paired with police-interaction vignettes, composites constructed by Black relative to White individuals evoked greater “fight-or-flight” behavioral intentions and anxiety, both of which are associated with threat (LeDoux & Pine, 2016).

Additionally, despite separately operationalizing negativity versus danger, limitations arise in our differential assessments of threat versus valence. High police-danger and police-bad correlations in Study 1 (*r* = 0.73) suggested that we were not capturing these associations in a vacuum. Less ambiguity was demonstrated in Study 2 when danger and negativity were pitted against each other head-to-head. Dangerous responses on our MPs might imply an association of police with nearby danger (i.e., crime or criminal suspects) and not necessarily indicate evaluations of police as survival threats themselves. Yet uniformed non-police that are also associated with nearby danger (firefighters) led to more safe versus dangerous and good versus bad evaluations in Study 1, and, importantly, more negative versus dangerous evaluations in Study 2. Therefore, we do not consider the police-danger associations to be a result of an external danger association.

### Future directions

A key focus of future work should consider the physiological and automatic defensive behavioral implications of police-danger associations. Physiological indices of threat (e.g., heart-rate variability, startle eye-blink) may speak to whether police activate the defense cascade, potentially offering an additional measure to distinguish threat from valence (March et al., 2017). Likewise, the automatic behavioral implications of threat during encounters with the police could be approximated using tasks that assess behavior resulting from rapid evaluations (e.g., approach/avoid). These responses are of particular relevance to police-civilian interaction. If police evoke the defense cascade and reflexive freeze or active avoidance behaviors (i.e., fight, flight, or freeze), such findings could speak to certain instances of civilian non-compliance.

## Conclusion

The present work explored the role of civilians’ implicit police-threat and police-valence evaluations in underlying explicit views of the police. Across two studies, we found evidence that the extent to which civilians implicitly associate the police with danger predicts people’s viewing police as more negative. The present findings offer insights to the cognitive fallout of systemic police violence.

## Supplementary Information

**Additional file 1**. Supplemental materials including the Perceptions of Police Scale, all prime stimuli used, the means and standard deviations for all prime conditions in Study 1, and zero-order correlations for all variables in Studies 1 and 2.

## Data Availability

All data and analysis code from the present study are available on request from Vincenzo Olivett, olivett@psy.fsu.edu.

## Abbreviations

MP: Misattribution procedure(s)
POPS: Perceptions of police scale

## Footnotes

1 The current work reports data collected from a sample of all White participants. We expected that White participants would provide a conservative test of the idea of police-danger driving explicit perceptions of police as Whites may not typically see themselves as victims of police violence based on recent media focus. Although these results should theoretically extend to other races and ethnicities, we acknowledge this is as a limitation in the General Discussion ripe for future exploration.

2 This task also contained non-hypothesis relevant trials that contained positive (e.g., puppy), negative (e.g., cockroach), or threatening (e.g., spider) objects (Lang et al., 2008). These trials were included to test a secondary question unrelated to the current work. Interested readers can find the mean responses to those trials in the Supplementary Materials.
