# Supplementary material for: White civilians’ implicit danger evaluation of police officers underlies explicit perception of police
Source: Cogn Res Princ Implic. 2021 Dec 20;6:81. doi: 10.1186/s41235-021-00343-9 (PMC8688646; doi:10.1186/s41235-021-00343-9)
Supplement: Supplementary file 1 — Additional file 1. Supplemental materials including the Perceptions of Police Scale, all prime stimuli used, the means and standard deviations for all prime conditions in Study 1, and zero-order correlations for all variables in Studies 1 and 2. [file 41235_2021_343_MOESM1_ESM.docx]

**Supplemental Materials**

**Prime Condition Stimuli**


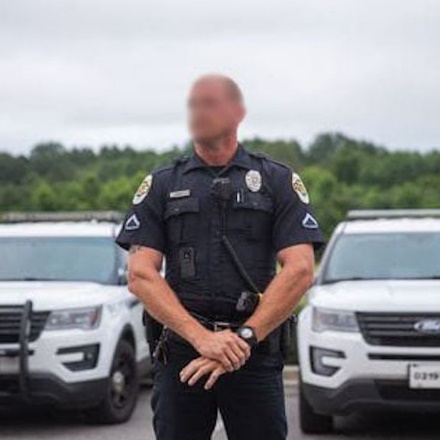

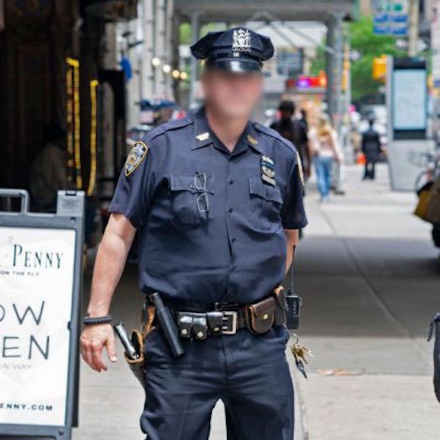

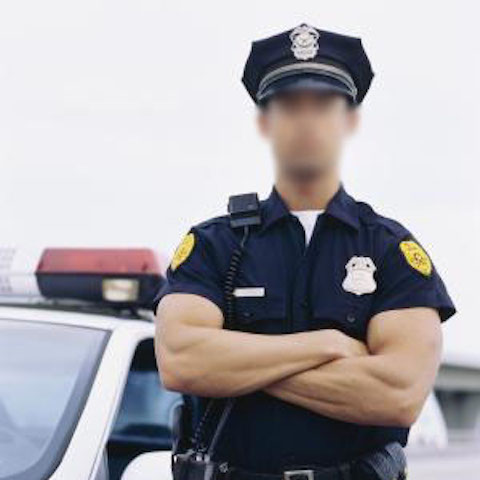

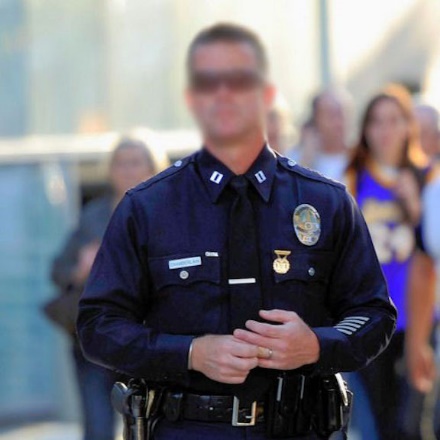

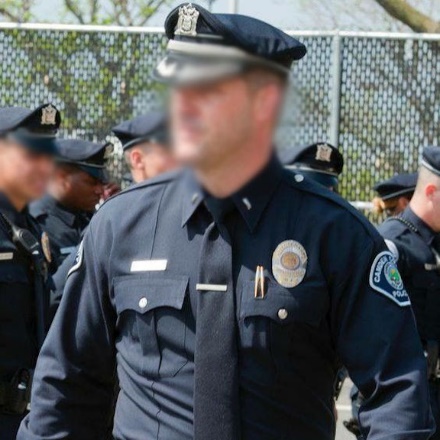

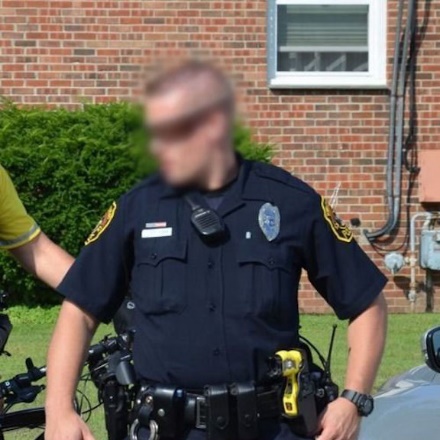

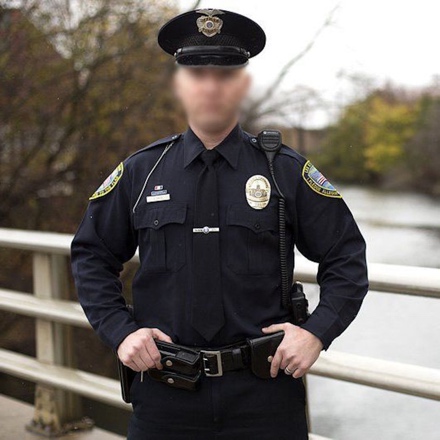

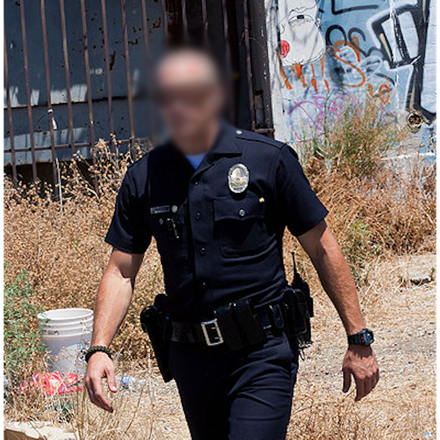

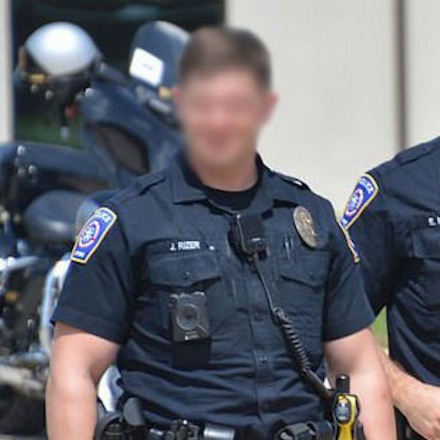

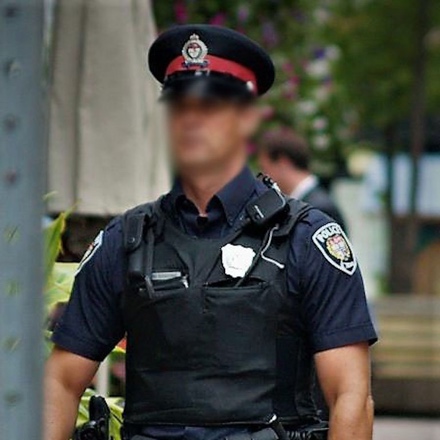
*Study 1 Police*

*
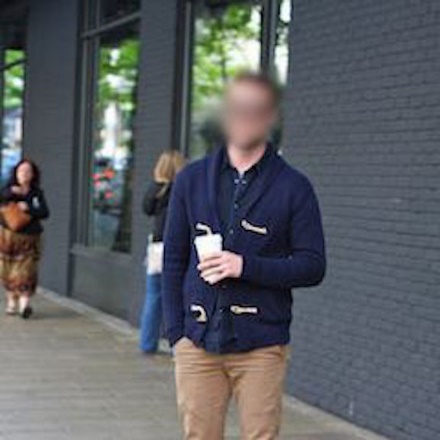

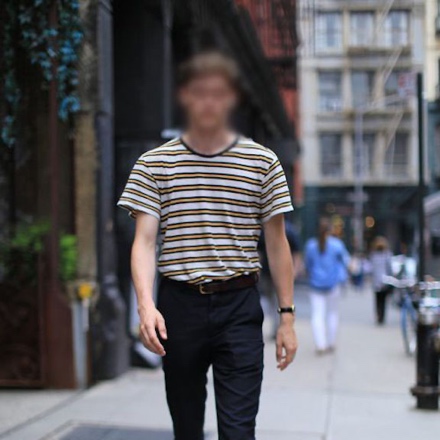

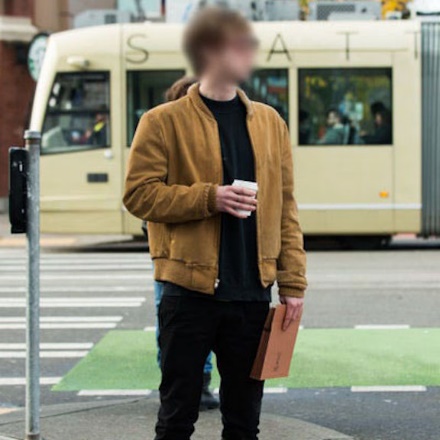

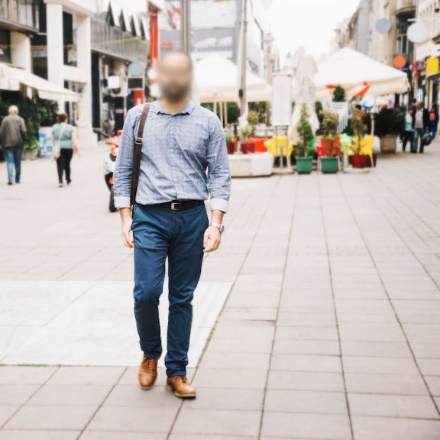

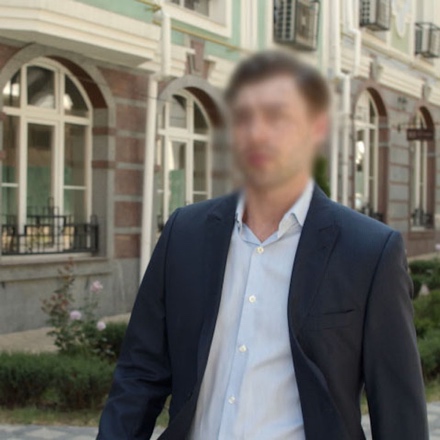

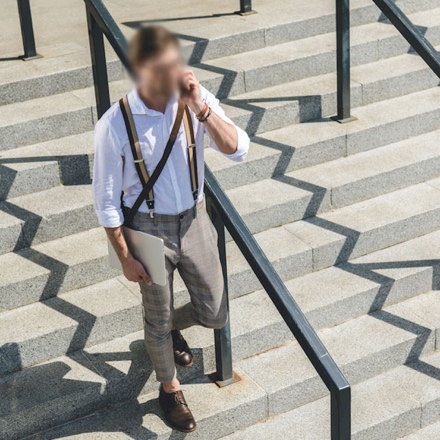

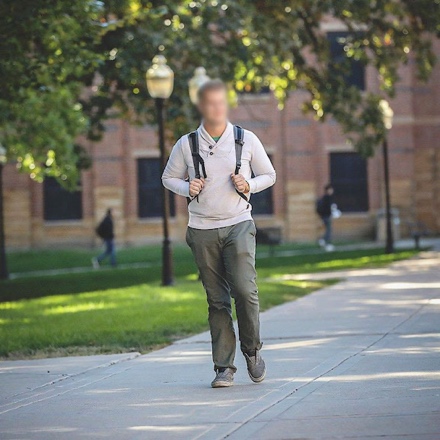

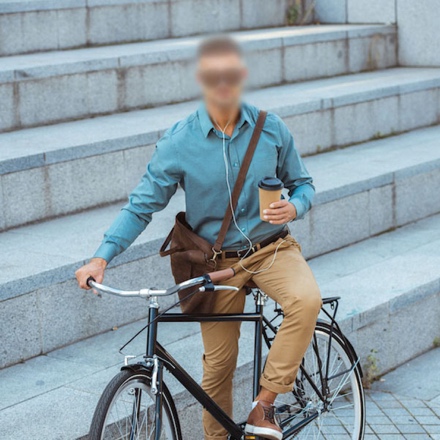

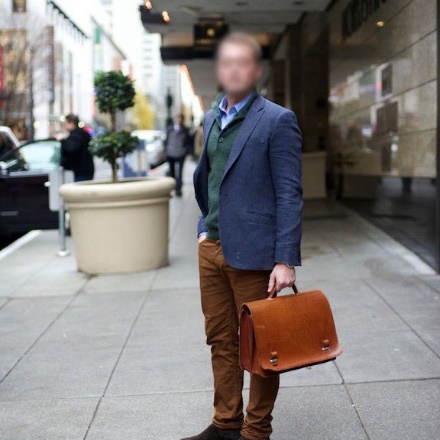

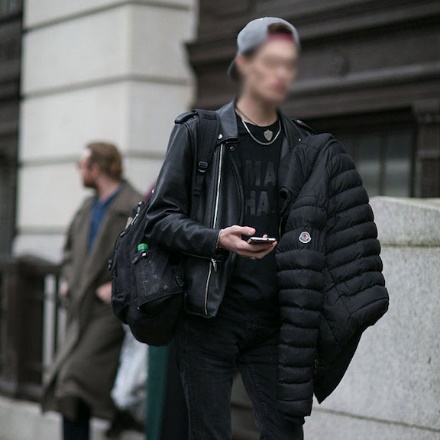
Study 1 Civilians*

*
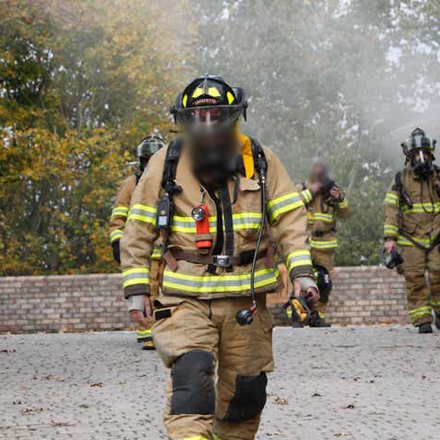

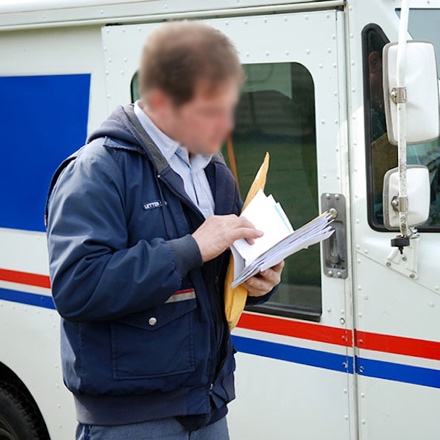

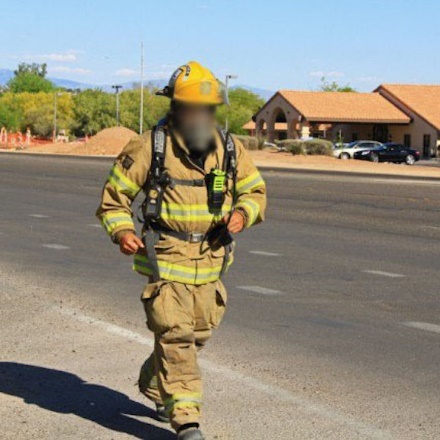

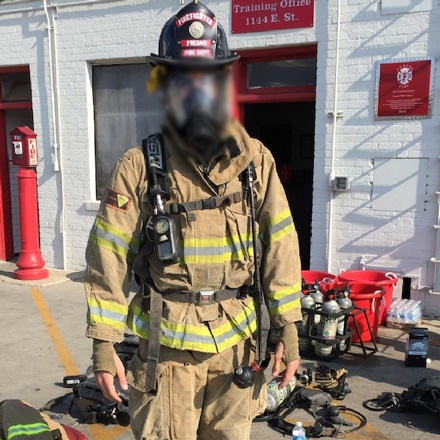

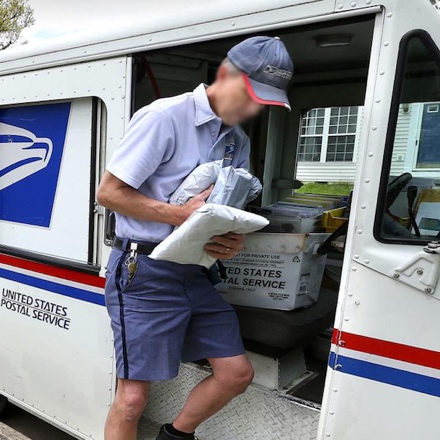

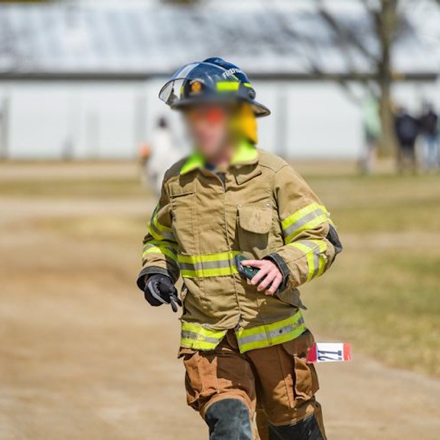

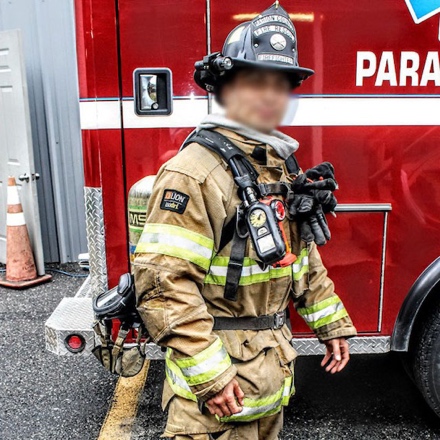

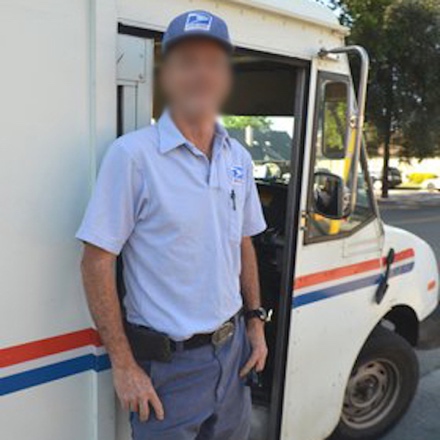

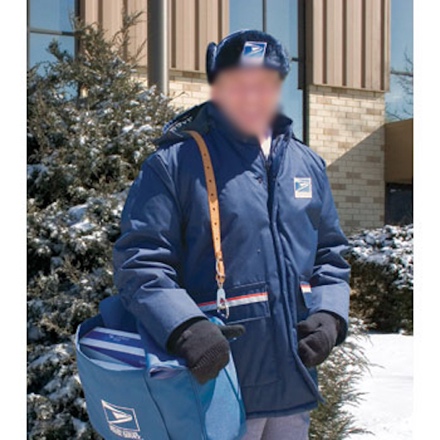

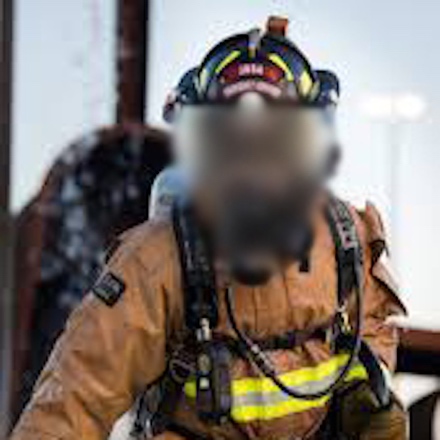
Study 1 Non-Police Uniformed Professionals*


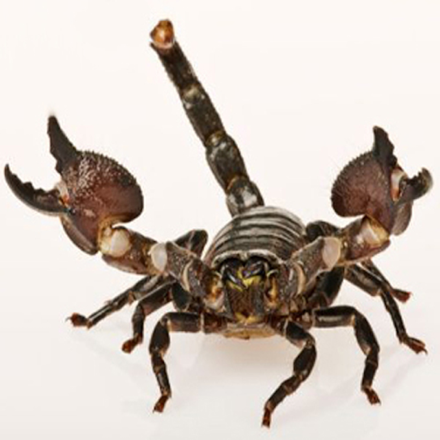

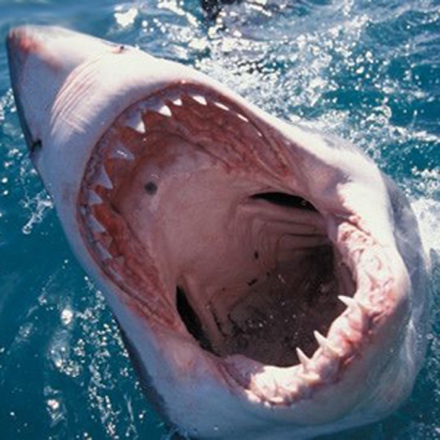

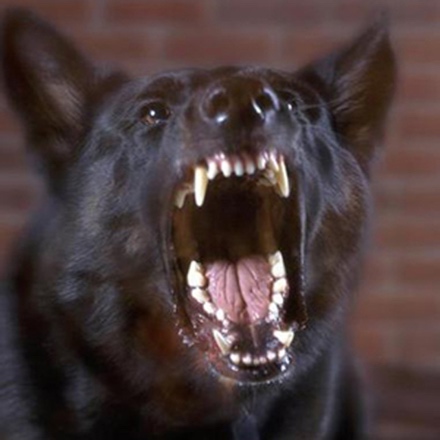

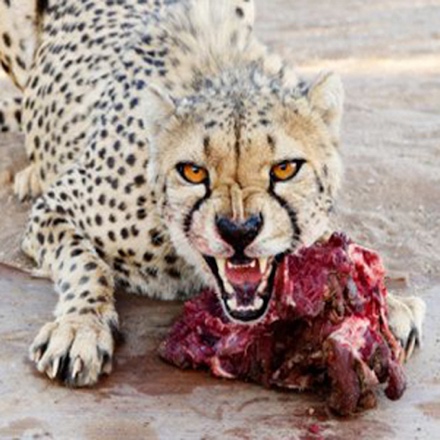

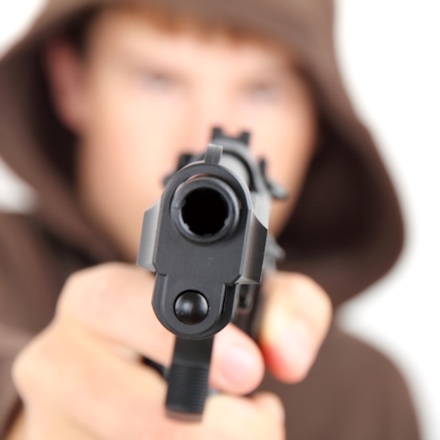

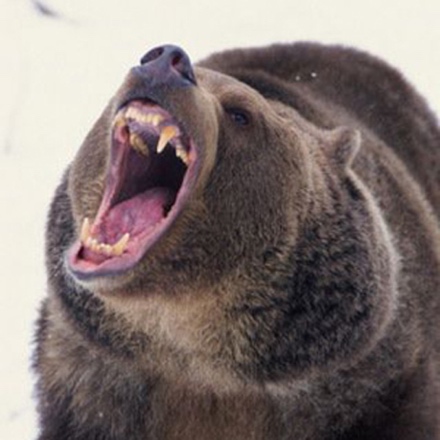

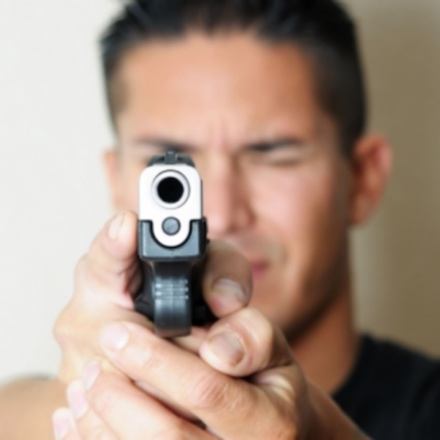

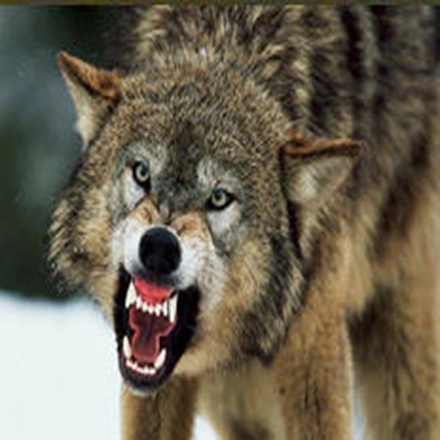

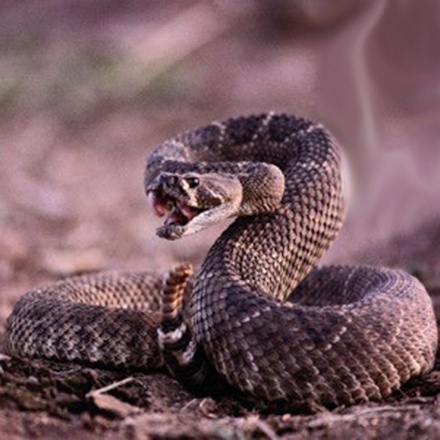

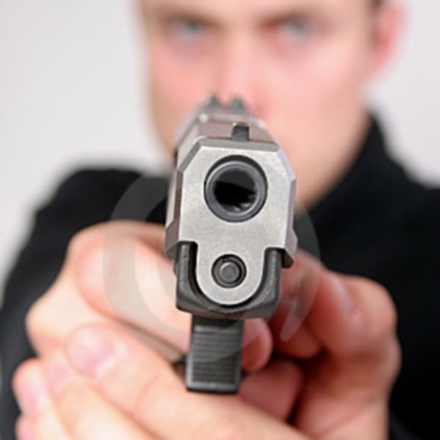
*Study 1 Threat*


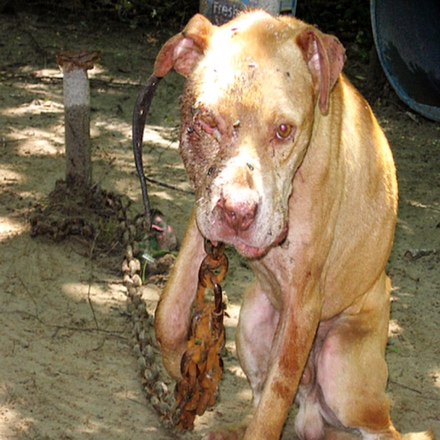

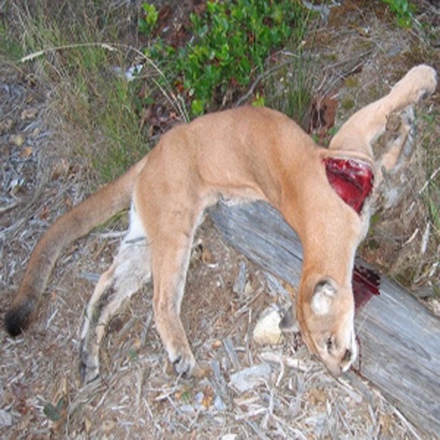

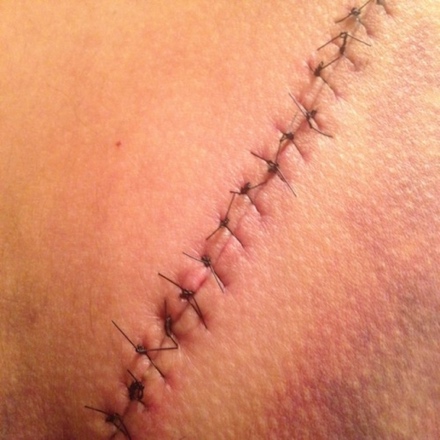

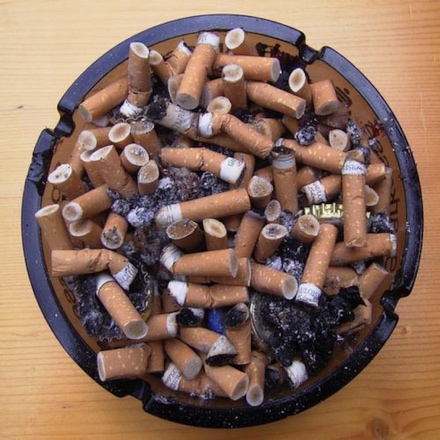

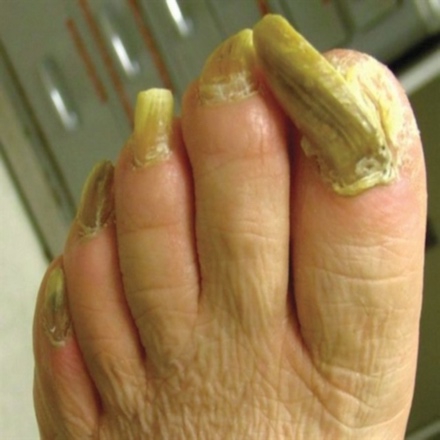

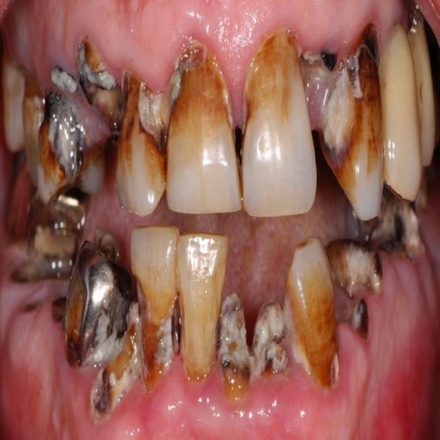

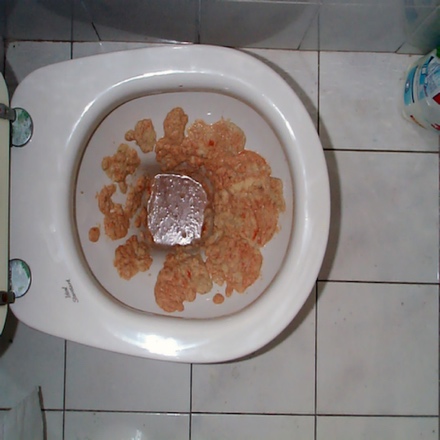

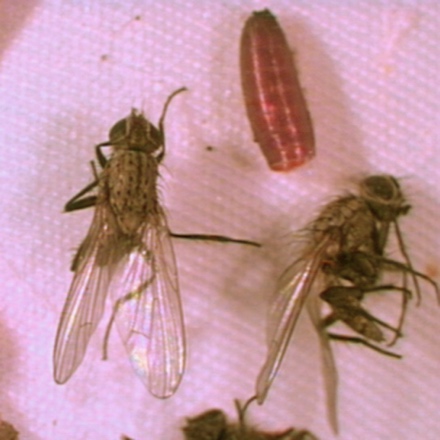

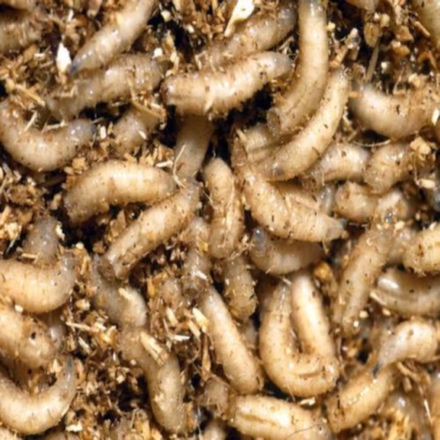

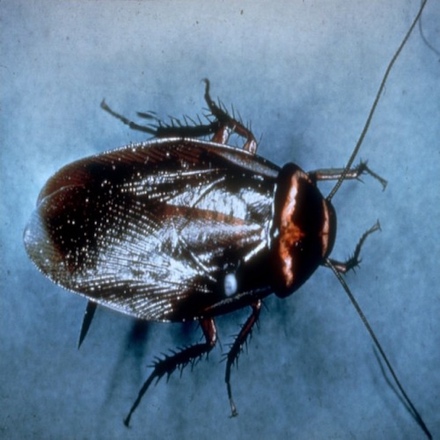
*Study 1 Negative*


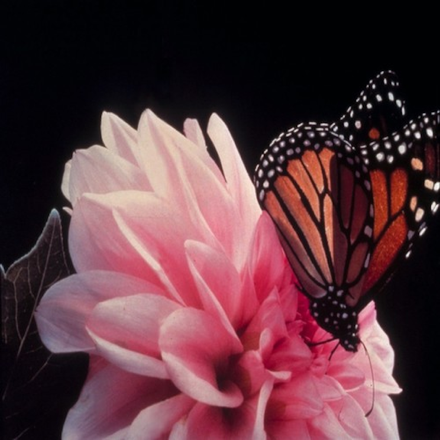

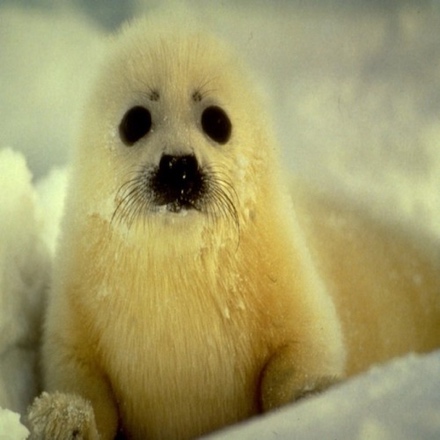

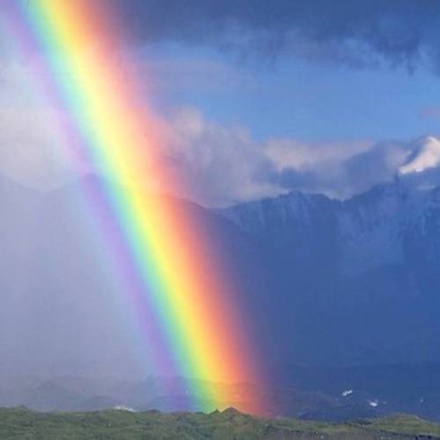

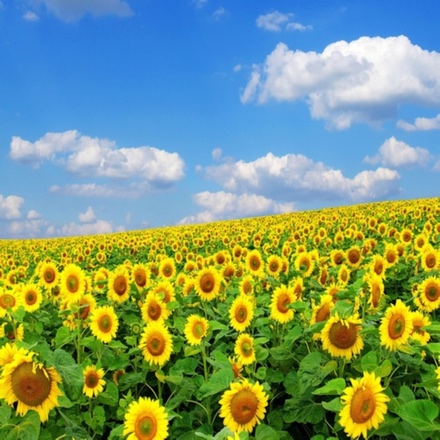

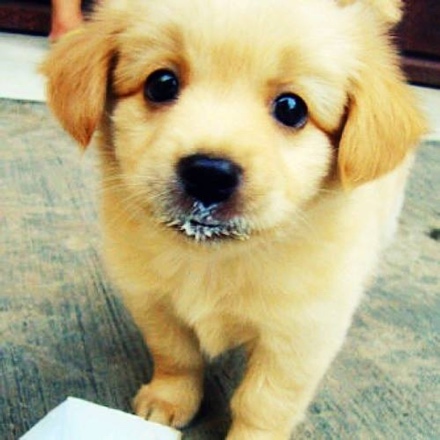

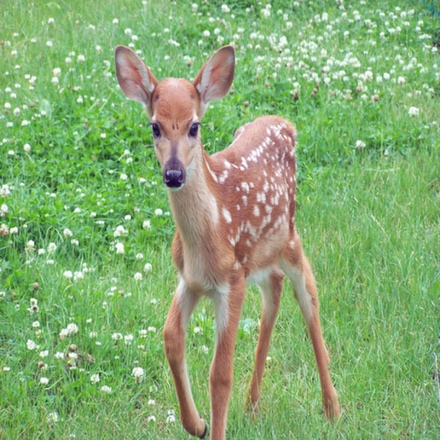

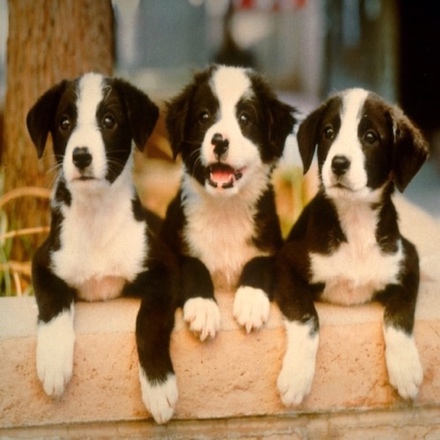

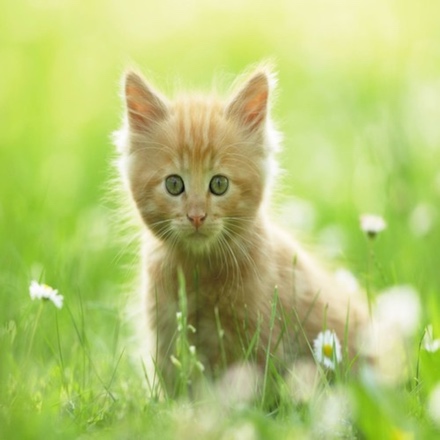

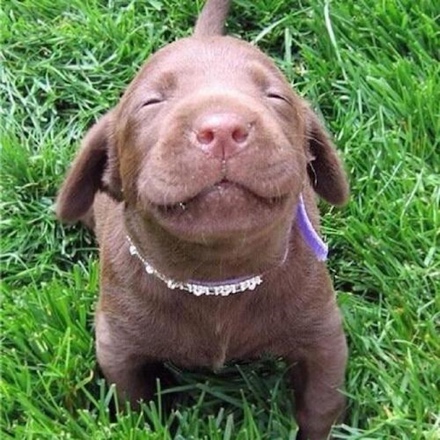

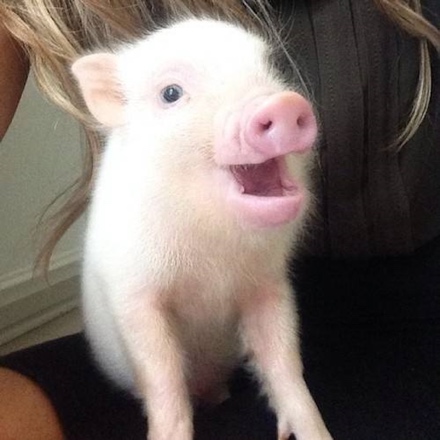
*Study 1 Positive*


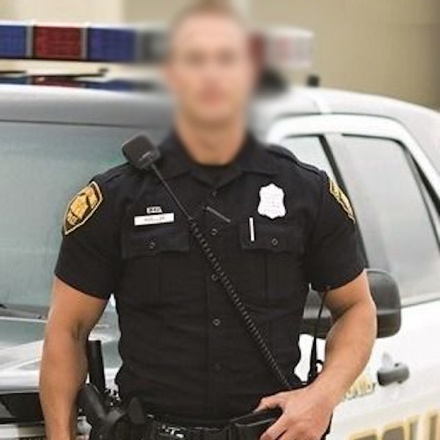

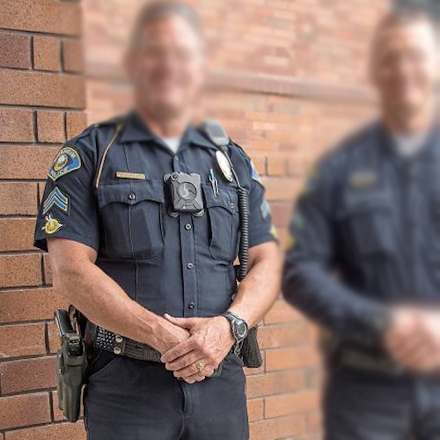

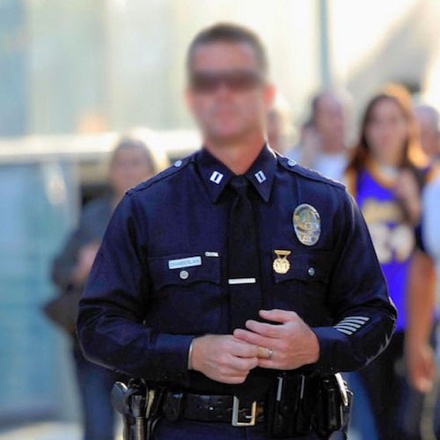

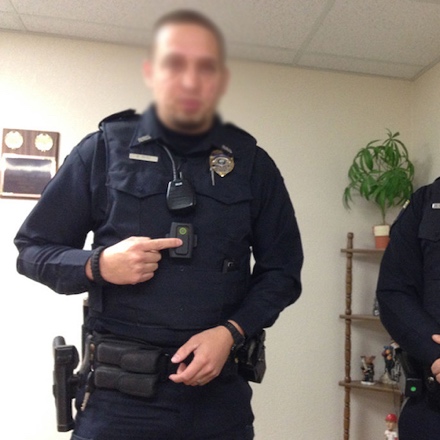

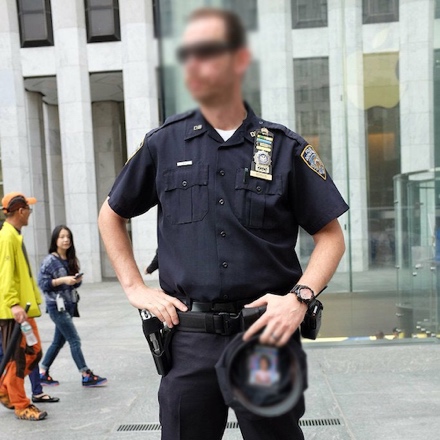
*Study 2 Armed Police*


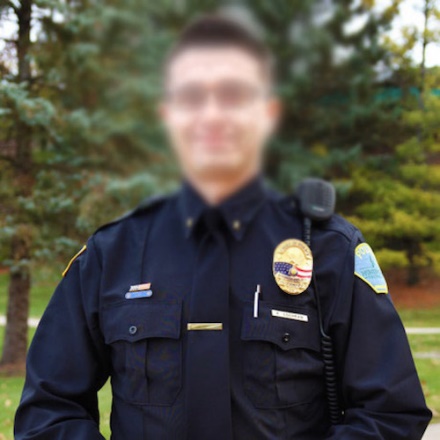

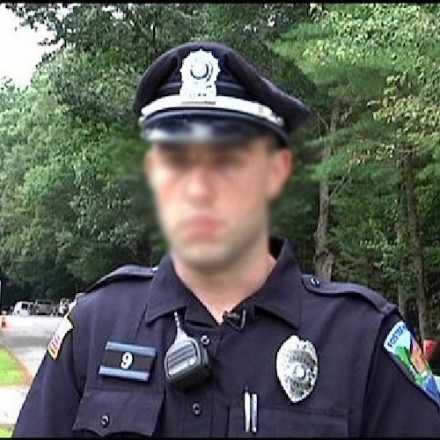

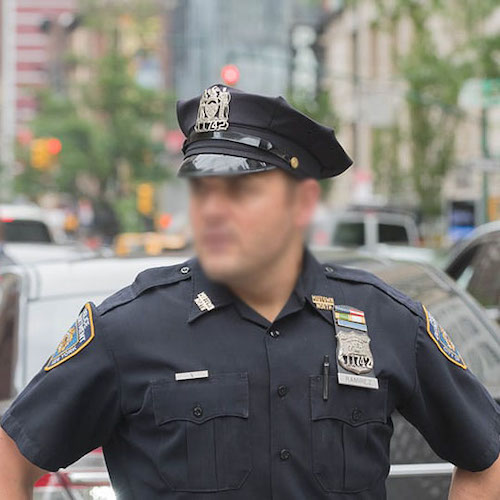

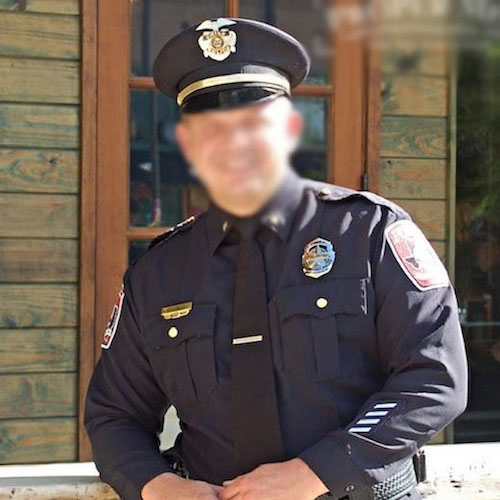

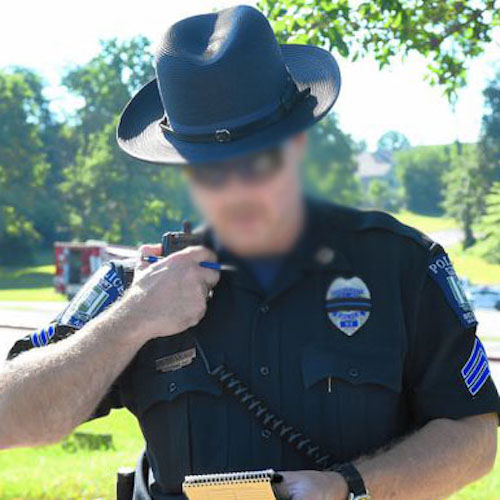
*Study 2 Unarmed Police*


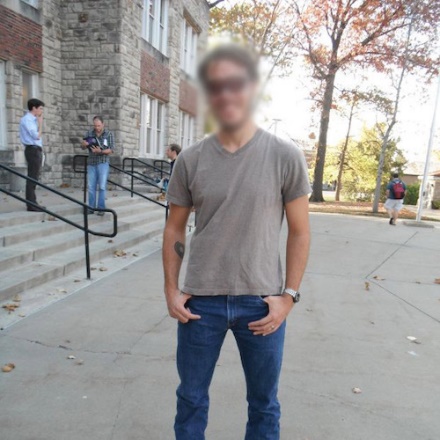
*Study 2 Civilians*


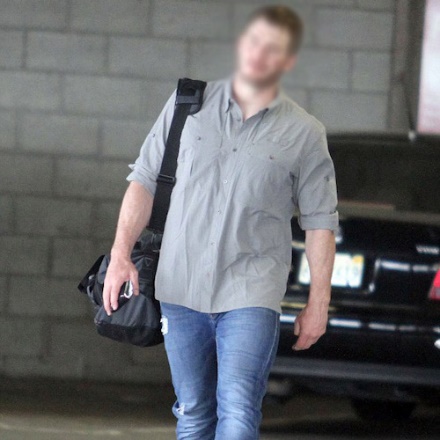

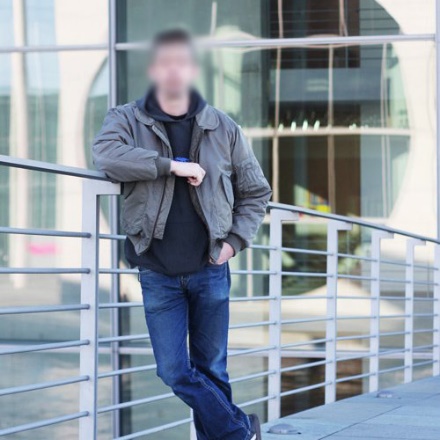
*
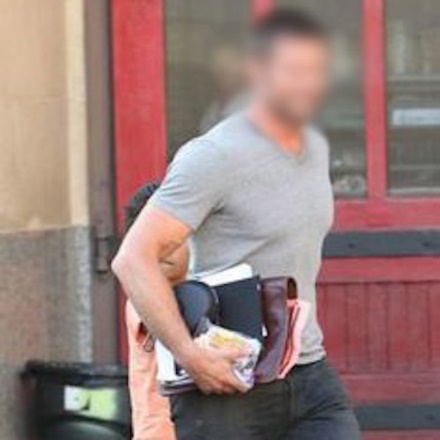

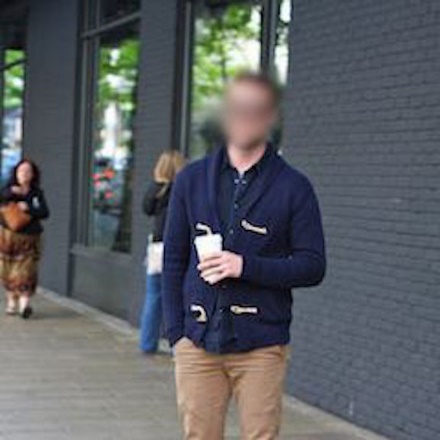

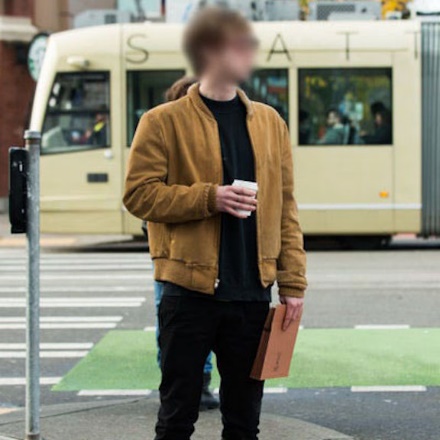

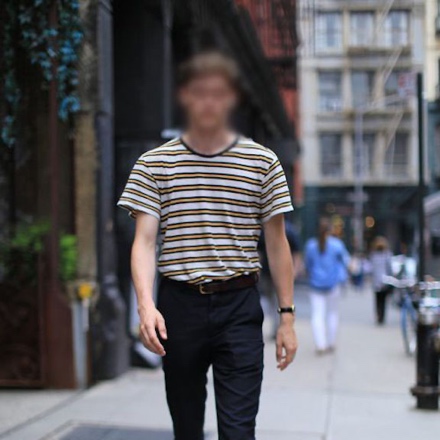

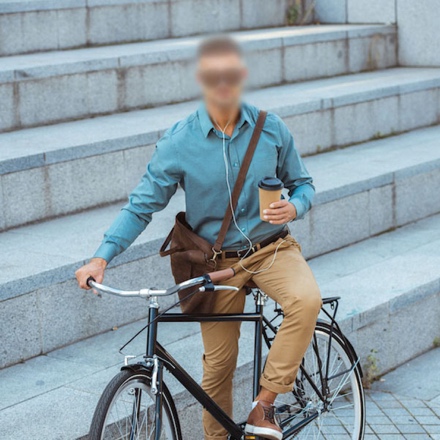

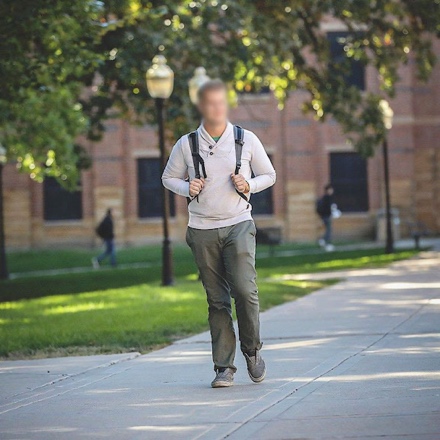

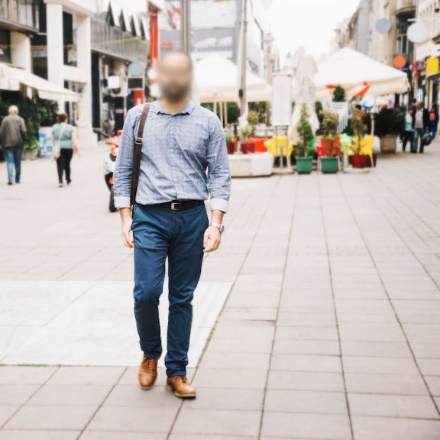
*


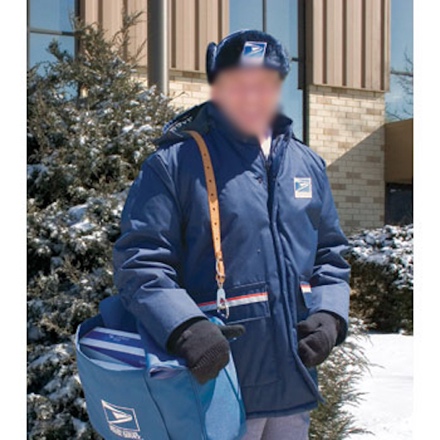

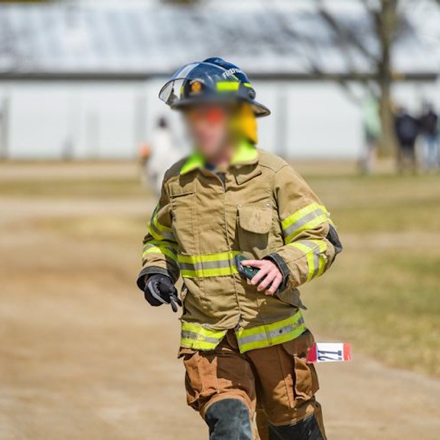

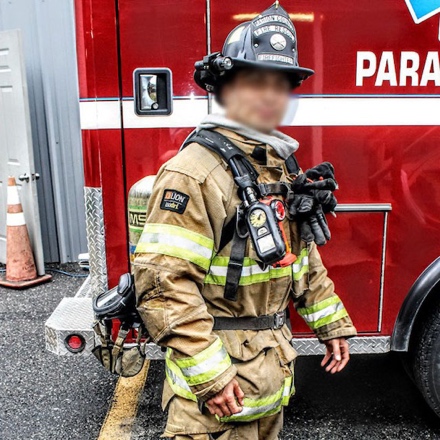

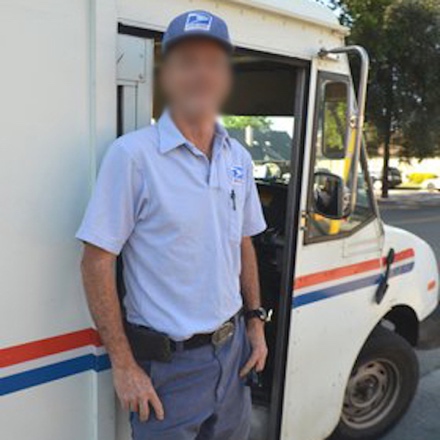

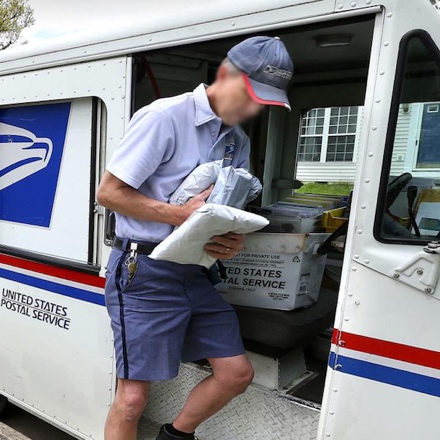

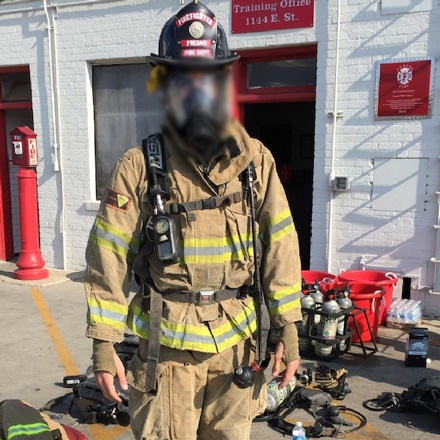

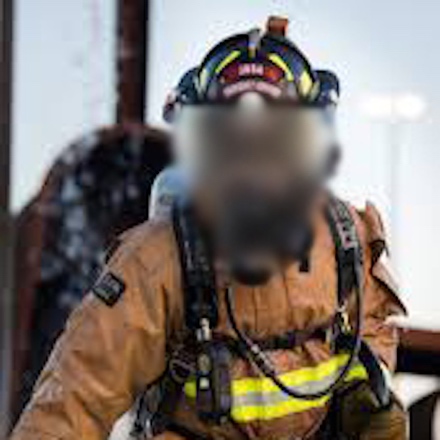

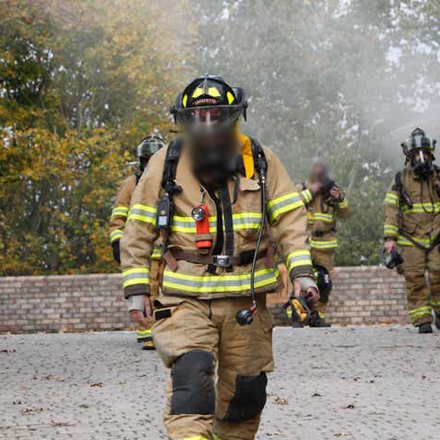

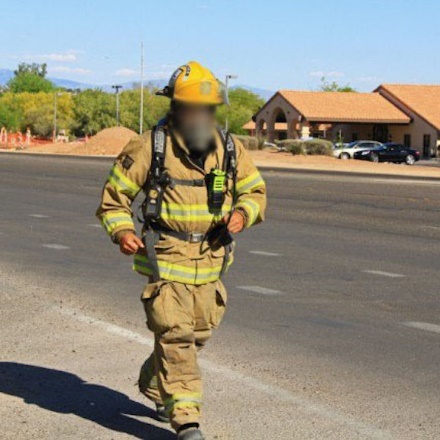

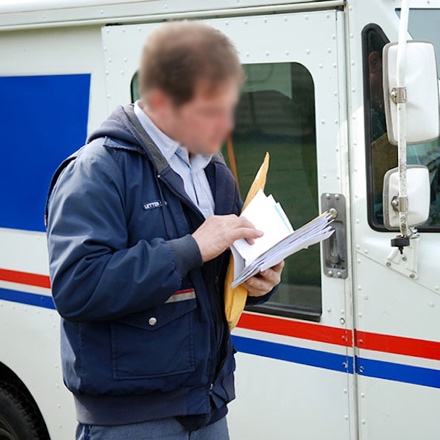
*Study 2 Uniform*

**Perceptions of Police Scale (POPS; Nadal & Davidoff, 2015)**

“Please provide the extent to which you agree or disagree with the following statement:”

(Scale: 1 = “strongly disagree”, 2 = “disagree”, 3 = “neither agree nor disagree”, 4 = “agree”, 5 = “strongly agree”)

1. Police officers are friendly
2. Police officers protect me
3. Police officers treat all people fairly
4. I like the police
5. The police are good people
6. The police do not discriminate
7. The police provide safety
8. The police are helpful
9. The police are trustworthy
10. The police are reliable
11. Police officers are unbiased
12. Police officers care about my community

| *Table S1*. Zero-order Correlations between all Variables in Study 1 | | | | | | | | | | | | | | |  |
| --- | --- | --- | --- | --- | --- | --- | --- | --- | --- | --- | --- | --- | --- | --- | --- |
|  | **POPS** | **Police-T** | **Civilian-T** | **Uniform-T** | **Threat-T** | **Negative-T** | **Positive-T** | **Police-V** | **Civilian-V** | **Uniform-V** | **Threat-V** | **Negative-V** | **Positive-V** | **Pol Id** | |
| **POPS** | - |  |  |  |  |  |  |  |  |  |  |  |  |  | |
| **Police-T** | -.65*** | - |  |  |  |  |  |  |  |  |  |  |  |  | |
| **Civilian-T** | -.018 | .18* | - |  |  |  |  |  |  |  |  |  |  |  | |
| **Uniform-T** | -.073 | .23* | .55*** | - |  |  |  |  |  |  |  |  |  |  | |
| **Threat-T** | -.056 | .012 | -.59*** | -.51*** | - |  |  |  |  |  |  |  |  |  | |
| **Negative-T** | .068 | -.016 | -.32*** | -.37*** | .67*** | - |  |  |  |  |  |  |  |  | |
| **Positive-T** | .19* | -.004 | .57*** | .61*** | -.61*** | -.46*** | - |  |  |  |  |  |  |  | |
| **Police-V** | -.55*** | .73*** | .077 | .15 | .009 | -.024 | -.023 | - |  |  |  |  |  |  | |
| **Civilian-V** | -.057 | .048 | .46*** | .29** | -.47*** | -.27* | .39*** | .14 | - |  |  |  |  |  | |
| **Uniform-V** | -.097 | .13 | .41*** | .49*** | -.47*** | -.37*** | .53*** | .21* | .48*** | - |  |  |  |  | |
| **Threat-V** | .098 | -.069 | -.47*** | -.34*** | .45*** | .38*** | -.37*** | .0003 | -.39*** | -.42*** | - |  |  |  | |
| **Negative-V** | .016 | -.098 | -.41*** | -.37*** | .35*** | .36*** | -.32** | -.012 | -38*** | -.30** | 0.61*** | - |  |  | |
| **Positive-V** | -.024 | -.012 | .37*** | .35*** | -.38*** | -.29** | .38*** | .057 | .47*** | .55*** | -.61*** | -.44*** | - |  | |
| **Pol Id** | -.76*** | .47*** | .023 | 0.11 | 0.029 | -.03 | -.10 | .42*** | .083 | .12 | -.098 | .071 | 0.029 | - | |
| Note: * indicates significance at the <.05 level; ** indicates significance at the <.01 level; *** indicates significance at the <.001 level.  T = Threat (high values indicate more “danger” vs. “safe” evaluations). Ex: Police-T are danger relative to safe responses towards targets following police primes on the threat MP.  V = Valence (high values indicate more “bad” vs. “good” evaluations). Ex: Police-V are bad relative to good responses towards targets following police primes on the valence MP.  POPS = Perceptions of Police Scale (1 = negative views, 3 = neutral views, 5 = positive views)  Pol Id = Mean-centered political orientation (larger vales represent more liberal vs. conservative | | | | | | | | | | | | | | |  |

*Table S2.* Means and standard deviations for all prime categories in Study 1

| **Prime Category** | **Mean** | **SD** |
| --- | --- | --- |
| **Police Good/Bad** | 0.41 | 0.32 |
| **Civilian Good/Bad** | 0.27 | 0.22 |
| **Uniform Good/Bad** | 0.24 | 0.23 |
| **Threat Good/Bad** | 0.72 | 0.27 |
| **Negative Good/Bad** | 0.71 | 0.22 |
| **Positive Good/Bad** | 0.19 | 0.20 |
| **Police Safe/Dangerous** | 0.42 | 0.34 |
| **Civilian Safe/Dangerous** | 0.28 | 0.24 |
| **Uniform Safe/Dangerous** | 0.25 | 0.22 |
| **Threat Safe/Dangerous** | 0.78 | 0.22 |
| **Negative Safe/Dangerous** | 0.72 | 0.22 |
| **Positive Safe/Dangerous** | 0.19 | 0.29 |

*Table S3.* Zero-order Correlations between all Variables in Study 2

|  | **POPS** | **Police Eval** | **Civilian Eval** | **Uniform Eval** | **Pol Id** |
| --- | --- | --- | --- | --- | --- |
| **POPS** | - |  |  |  |  |
| **Police Eval** | -.34* | - |  |  |  |
| **Civilian Eval** | .094 | -.22* | - |  |  |
| **Uniform Eval** | -.088 | .13 | .36* | - |  |
| **Pol Id** | -.67*** | .16 | .072 | .004 | - |
| Note: * indicates significance at the <.05 level; ** indicates significance at the <.01 level; *** indicates significance at the <.001 level  Eval = Threat vs. valence response (high values indicate more “danger” vs. “negative” evaluations).Ex: Police evals are danger relative to negative responses towards targets following police primes on the threat MP.  POPS = Perceptions of Police Scale (1 = negative views, 3 = neutral views, 5 = positive views)  Pol Id = Mean-centered political orientation (larger vales represent more liberal vs. conservative | | | | | |
